# Retinal Oxygen Delivery, Metabolism and Extraction Fraction and Retinal Thickness Immediately Following an Interval of Ophthalmic Vessel Occlusion in Rats

**DOI:** 10.1038/s41598-019-44250-y

**Published:** 2019-05-30

**Authors:** Norman P. Blair, Michael R. Tan, Anthony E. Felder, Mahnaz Shahidi

**Affiliations:** 1 Department of Ophthalmology and Visual Sciences, University of Illinois at Chicago, Chicago, USA; 2 Richard and Loan Hill Department of Bioengineering, University of Illinois at Chicago, Chicago, USA; 3 Department of Ophthalmology, University of Southern California, Los Angeles, USA

**Keywords:** Retinal diseases, Blood flow

## Abstract

Limited knowledge is currently available about alterations of retinal blood flow (F), oxygen delivery (DO_2_), oxygen metabolism (MO_2_), oxygen extraction fraction (OEF), or thickness after the ophthalmic blood vessels have been closed for a substantial interval and then reopened. We ligated the ophthalmic vessels for 120 minutes in one eye of 17 rats, and measured these variables within 20 minutes after release of the ligature in the 10 rats which had immediate reflow. F, DO_2_ and MO_2_ were 5.2 ± 3.1 μL/min, 428 ± 271 nL O_2_/min, and 234 ± 133 nL O_2_/min, respectively, that is, to 58%, 46% and 60% of values obtained from normal fellow eyes (P < 0.004). OEF was 0.65 ± 0.23, 148% of normal (P = 0.03). Inner and total retinal thicknesses were 195 ± 24 and 293 ± 20 μm, respectively, 117% and 114% of normal, and inversely related to MO_2_ (P ≤ 0.02). These results reflect how much energy is available to the retina immediately after an interval of nonperfusion for 120 minutes. Thus, they elucidate aspects of the pathophysiology of nonperfusion retinal injury and may improve therapy in patients with retinal artery or ophthalmic artery obstructions.

## Introduction

Inner retinal ischemia is a major pathophysiologic factor in retinal vascular occlusions, diabetic retinopathy^1–4^, sickle cell retinopathy^5^, and has been implicated in glaucoma^6–10^. Much important information about ischemic retinal injury, and pharmacological modification of it, has been obtained from animal models of retinal ischemia. In most of these models, retinal blood flow is interrupted, and, since the baseline oxygen metabolic rate normally is high^11,12^ and oxygen stores are minimal^13^, oxygen metabolism rapidly ceases. In many of the models, obstruction of the retinal blood supply can be reversed after some time period, for example, by release of a ligature or by normalizing the intraocular pressure^14–23^. In that case, improvement of retinal blood flow and inner retinal oxygen metabolism can occur. However, upon reflow, reperfusion injury is known to develop in neural tissue^24,25^, and it may be difficult to separate the effects of hypoperfusion and reperfusion on the ultimate tissue outcome. In fact, reports on retinal ischemia often equivocate on this point and describe their observations as being on ischemia-reperfusion injury.

In these reperfusion models, knowledge is limited about the improvement of blood flow and oxygen metabolism after reversible retinal ischemia because these outcomes usually have not been measured. However, post-ischemic hyperemia has been observed in the retina of cats and rats after 60 minutes or less of ischemia^26–29^. On the other hand, incomplete normalization of retinal blood flow by obstruction of the microvasculature with leukocytes after ischemia has been reported^30,31^, consistent with the “no reflow phenomenon” observed in the brain^24,32–35^. Since the eventual outcome following severe ischemia is known to be highly dependent on the duration of ischemia between about 60 and 120 minutes (i.e., improvement is nearly complete with durations less than 60 minutes, and improvement is minimal at durations beyond 120 minutes)^16,18,20,22,23,36^, it is difficult to extrapolate from the above results how inner retinal blood flow would improve immediately after severe retinal ischemia at durations toward the end of this interval. Furthermore, essentially no information is available about the improvement of oxygen delivery and metabolism shortly after retinal ischemia. It is known, however, that ischemia can result in anatomic and physiologic injury to mitochondria that limits resumption of normal oxygen metabolism^37–39^.

Since retinal function requires energy, it is dependent on a continuous supply of oxygen and nutrients. Thus, the ideal therapy for severely impaired blood flow beyond the 60 minute duration would appear to be to 1) re-establish perfusion and oxygenation, and 2) suppress reperfusion injury. Consequently, it is important to characterize how blood flow and oxygen metabolism improve as part of the overall enterprise to maximize retinal survival after an ischemic insult. Since there is evidence for post-ischemic hyperemia, we hypothesized that immediately after an interval in which retinal and choroidal blood flow had been eliminated by ophthalmic vessel occlusion (OVO)^20^ the improvement of blood flow, and, consequently, oxygen delivery, would be greater than that of oxygen metabolism. We tested this hypothesis using methods we have developed previously to measure inner retinal blood flow (F), oxygen delivery (DO_2_), oxygen metabolism (MO_2_), and the oxygen extraction fraction (OEF) in rats^21,40^. In addition, since the retina thickens after ischemia^41–43^ and there is a lack of information about any role of MO_2_ in this, we tested the hypothesis that there is a relationship between retinal thickening and the reduction in MO_2_ immediately following an interval of nonperfusion.

## Results

### Retinal nonperfusion with prompt reperfusion

The retina reperfused immediately in 10 rats after 120 minutes of nonperfusion. Table 1 shows retinal arterial and venous PO_2_ (PO_2A_ and PO_2V_, respectively), arterial and venous diameter (D_A_ and D_V_, respectively), venous velocity (V), and F measurements in these rats. PO_2A_ and PO_2V_ were lower in eyes with OVO compared to those in fellow eyes (P ≤ 0.01). D_A_ was lower in eyes with OVO relative to that in their fellow eyes (P < 0.001), while the difference in D_V_ between eyes with OVO and their fellow eyes was not statistically significant (P = 0.09). Measurements of V and F were reduced in eyes with OVO compared to those in fellow eyes (P ≤ 0.002). F in eyes with OVO improved to 58% of that in their fellow eyes.

**Table 1:** Retinal Arterial and Venous Oxygen Tension (PO_2A_ and PO_2V_, respectively), Arterial and Venous Diameter (D_A_ and D_V_, respectively), Venous Blood Velocity (V) and Venous Blood Flow (F) in Rat Eyes with Ophthalmic Vessel Occlusion (OVO) and Fellow Eyes after 2 Hours of Nonperfusion (N = 10). Statistically significant P values in bold.

	Eyes with OVO (Mean ± SD)	Fellow Eyes (Mean ± SD)	P value
PO_2A_ (mm Hg)	37 ± 6	45 ± 7	**0.004**
PO_2V_ (mm Hg)	20 ± 9	31 ± 5	**0.01**
D_A_ (μm)	40 ± 6	47 ± 5	**<0.001**
D_V_ (μm)	60 ± 10	54 ± 4	0.09
V (mm/s)	4.0 ± 2.0	9.7 ± 2.0	**<0.001**
F (μL/min)	5.2 ± 3.1	9.0 ± 2.1	**0.002**

Table 2 displays mean inner retinal DO_2_, MO_2_, and OEF measured after 120 minutes of nonperfusion and prompt reperfusion. DO_2_ was lower in eyes with OVO (428 ± 271 nL O_2_/min) compared to that in their fellow eyes (924 ± 284 nL O_2_/min) (P = 0.001), an improvement to only 46% of that in the fellow eye. Similarly, MO_2_ was lower in eyes with OVO (234 ± 133 nL O_2_/min) relative to that in their fellow eyes (389 ± 87 nL O_2_/min) (P = 0.004), improving to 60% of what was present in the fellow eye. OEF was higher in eyes with OVO (0.65 ± 0.23) compared to that in their fellow eyes (0.44 ± 0.08) (P = 0.03), 48% greater than in fellow eyes.

**Table 2:** Retinal Oxygen Metabolism (MO_2_), Oxygen Delivery (DO_2_) and Oxygen Extraction Fraction (OEF) in Rat Eyes with Ophthalmic Vessel Occlusion (OVO) and Fellow Eyes after 2 Hours of Nonperfusion (N = 10). Statistically significant P values in bold.

	Eyes with OVO (Mean ± SD)	Fellow Eyes (Mean ± SD)	P value
DO_2_ (nL O_2_/min)	428 ± 271	924 ± 284	**0.001**
MO_2_ (nL O_2_/min)	234 ± 133	389 ± 87	**0.004**
OEF	0.65 ± 0.23	0.44 ± 0.08	**0.03**

Table 3 shows inner retinal thickness (IRT), outer retinal thickness (ORT) and total retinal thickness (TRT) measured after 120 minutes of nonperfusion with prompt reperfusion (thickness data were not available from one rat). IRT was higher in eyes with OVO (195 ± 24 μm) compared to that in the fellow eyes (166 ± 12 μm)(P = 0.009), an increase of 17% compared to that of the fellow eyes. ORT was not significantly different in eyes with OVO (98 ± 11 μm) compared to that in their fellow eyes (90 ± 7 μm) (P = 0.07). TRT was higher in eyes with OVO (293 ± 20 μM) compared to that in their fellow eyes (256 ± 13 μm) (P < 0.001), increasing by 14% compared to their fellow eyes.

**Table 3:** Inner Retinal Thickness (IRT), Outer Retinal Thickness (ORT), and Total Retinal Thickness (TRT) in Rat Eyes with Ophthalmic Vessel Occlusion (OVO) and Fellow Eyes after 2 Hours of Nonperfusion (N = 9). Statistically significant P values in bold.

	Eyes with OVO Mean ± SD)	Fellow Eyes (Mean ± SD)	P value
IRT (μm)	195 ± 24	166 ± 12	**0.009**
ORT (μm)	98 ± 11	90 ± 7	0.07
TRT (μm)	293 ± 20	256 ± 13	**<0.001**

**Figure 1:**
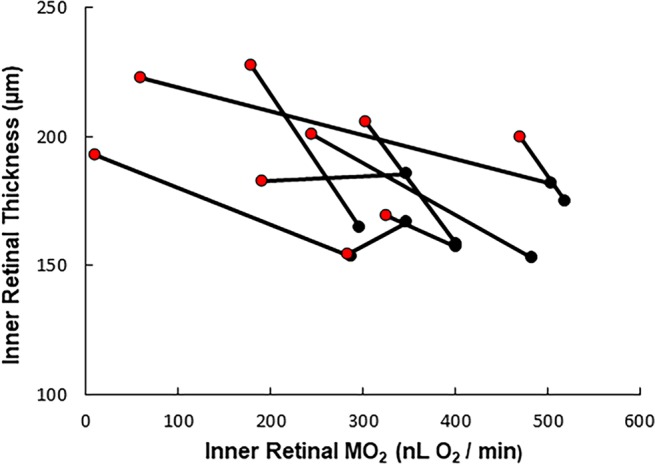
Relationship between inner retinal thickness and inner retinal oxygen metabolism (MO_2_) in rats with normal fellow and nonperfusion eyes. Data from the normal fellow eyes (black) are connected to the corresponding data obtained from the eyes immediately after 120 minutes of total ocular nonperfusion (red). An inverse linear relationship was found between increases in inner retinal thickness values and nonperfusion-induced reductions in MO_2_ values (β = −0.093 µm * min * nL O_2_^−1^, P = 0.019, N = 9).

**Figure 2:**
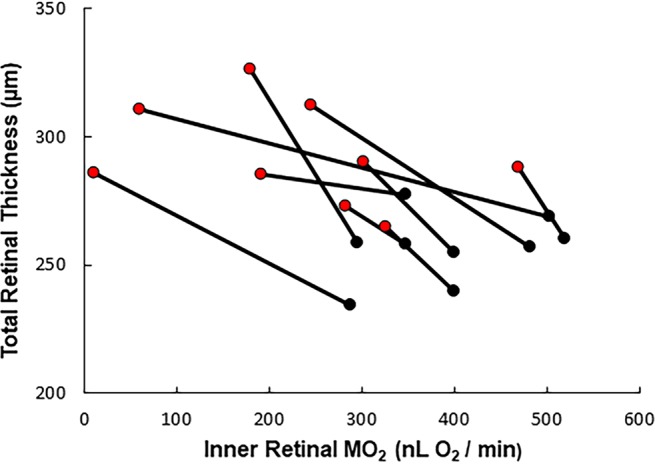
Relationship between total retinal thickness and inner retinal oxygen metabolism (MO_2_) in rats with normal fellow and nonperfusion eyes. Data from the normal fellow eyes (black) are connected to the corresponding data obtained from the eyes immediately after 120 minutes of total ocular nonperfusion (red). An inverse linear relationship was found between increases in total retinal thickness values and nonperfusion-induced reductions in MO_2_ values (β = −0.12 µm * nL O_2_^−1^ * min, P = 0.006, N = 9).

The mixed linear model revealed that IRT was linearly related to MO_2_ (β = −0.09 µm * nL O_2_^−1^ * min; P = 0.02, Fig. 1). In addition, a linear relationship between TRT and MO_2_ (β = −0.12 µm * nL O_2_^−1^ * min, P = 0.006, Fig. 2) was found. No significant relationship between ORT and MO_2_ was observed (P = 0.3).

### Retinal nonperfusion without prompt reperfusion

In seven of the 17 eyes undergoing OVO, reflow did not occur immediately. In five of these, reflow had not occurred by one hour after release of the ligature, which precluded blood flow measurements, and the experiments were concluded. We could not interpret the results in the other two eyes with confidence.

## Discussion

The present study had several major findings. First, we rejected the hypothesis that with prompt reperfusion after total ocular nonperfusion for a substantial period of time (at least 120 minutes) the improvement of F and, consequently, DO_2_ would be robust, exceeding that of MO_2_. Instead, with respect to the normal fellow eyes, the percentage improvement in F, and especially DO_2_, did not exceed that of MO_2_, the values being 58%, 46% and 60%, respectively, after release of the ligature. Of note, F, DO_2_ and MO_2_ values in fellow eyes were similar to those previously published in healthy control eyes^44^.

While post-ischemic hyperemia has been well documented after shorter durations of retinal ischemia^26–29^, we did not observe this phenomenon with 120 minutes of nonperfusion. Not only did F fail to completely improve immediately, but in seven of the 17 eyes there was no improvement immediately, and in five eyes no reflow had occurred by one hour after release of the ligature. This “no reflow phenomenon”^24,30,32–35,45^ occurs particularly after substantial periods in which F is completely interrupted. It appears to result from small vessel obstruction by leukocytes^30,31,35^, erythrocytes^34^, microthrombi^33^, pericyte constriction^45^, as well as other possible contributing factors^34^. These factors will variably affect the level of improvement of F, and, therefore, quantifying a nonperfusion insult merely by the duration that the investigator imposes the occlusive intervention may often underestimate persisting, potentially injurious F reduction. Monitoring F after reversing the occlusive intervention is likely to better quantify the nonperfusion insult and reduce the variability of its effect on the outcome under study.

The second major finding was that DO_2_ did not improve as much as F within 20 minutes of reperfusion. In many situations DO_2_ is dominated by F because oxygen in the retinal arteries near the optic nerve has not been exposed significantly to oxygen extraction by the retinal tissue. In the current experiments, while F improved roughly to the same extent as MO_2_, this did not enable comparable improvement in oxygen availability because PO_2A_, and consequently the oxygen concentrations in the arteries (O_2A_) were reduced. We tentatively interpreted this to be the result of reduced arterial velocity, so that there was more time for oxygen to be extracted from the blood across the arterial walls prior to arriving at the measurement sites near the optic nerve head. A reduction in arterial blood velocity can be deduced from our data on V, D_A_ and D_V_. That DO_2_ did not improve as much as F indicates that there are limits to inferring DO_2_ from measurements of F alone.

The third major finding was that OEF was increased in OVO eyes after reperfusion as compared to fellow eyes. OEF is the ratio of MO_2_ to DO_2_ and, accordingly, quantifies the adequacy of oxygen supply relative to the tissue’s metabolic demand. Thus, the increase in OEF was a consequence of the fact that changes in MO_2_ exceeded those of DO_2_ after release of the ligature. OEF has been shown in the brain to be of prognostic value for predicting ischemic stroke^46–48^. We have previously reported increased retinal OEF in rats under systemic hypoxia and ischemia, and in mice with diabetes^21,49,50^. OEF may prove to have prognostic value in retinal ischemic conditions, as well.

The fourth major finding was that MO_2_ did not completely improve in the first 20 minutes after release of the ligature as compared to the level found in the normal fellow eye. We believe this to be the first report of MO_2_ after a substantial interval of retinal nonperfusion. If there had been complete recovery in the eyes with OVO, the value of MO_2_ would have been essentially the same as that in the normal fellow eyes (389 nL O_2_/min). However, this value is less than DO_2_ in the eyes with OVO (428 nL O_2_/min). This indicates that the incomplete improvement in MO_2_ only to 234 nL O_2_/min was not caused by the overall unavailability of oxygen to the tissue from the vasculature. There are at least two explanations for this. First, nonperfusion may have induced maldistribution of microvascular blood flow. Some inner retinal areas may have received low enough levels of flow that oxygen metabolism was limited to less than normal values for the corresponding tissue, whereas other areas may have received more flow than was needed to deliver the normal amount of oxygen to the corresponding tissue (that is, shunting). Second, nonperfusion may have injured the capacity of the tissue to utilize oxygen. We previously found that with short periods of graded reductions of retinal blood flow, the starving retina can draw out all of the delivered oxygen and raise OEF to its theoretical maximum value of one, that is, where MO_2_ equals DO_2_^21^. If the capacity to metabolize oxygen (and blood flow distribution) were normal, the reperfused retina would have been able to reach the normal MO_2_ value despite the reduced DO_2_ and produce an OEF of about 0.91. Thus, the MO_2_ of 234 nL O_2_/min and OEF of 0.65 after reperfusion are consistent with nonperfusion-induced injury to the metabolic machinery underlying MO_2_. Indeed, in the brain, and presumably in the comparable neural retina, ischemia has significant injurious effects on mitochondria. These include excitotoxic calcium entry, stimulation of degradative enzymes, production of reactive oxygen species, alterations in membrane permeability, increases in volume, loss of cytochrome c, impairment of energy generation and, eventually, cell death^37–39^. Further studies are needed to determine the relative contributions to our results of maldistribution of blood flow and impaired capacity to utilize oxygen.

While much remains to be discovered about MO_2_ in retinal ischemia, considerable information has been obtained about the rate of oxygen metabolism in the ischemic brain using positron emission tomography and MRI methods (these rates are referred to as rCMRO_2_ or OMI, which are similar to MO_2_). Threshold values for these variables have been found that correlate well with tissue that will not or has not survived, and they are superior to OEF and other parameters for this purpose^51–54^. It is reasonable to suppose that MO_2_ may also prove to have similar utility with further studies of its role in retinal ischemia. However, whether MO_2_ or OEF will prove to be the more valuable factor in retinal ischemia remains to be determined, and important roles for both MO_2_ (similar to oxygen extraction^55^ and uptake^56^) and OEF may be identified now that they can be evaluated in human disease^55–58^.

The fifth major finding was that we accepted the hypothesis that there is a relationship between retinal thickening and the reduction in MO_2_ immediately following an interval of nonperfusion. The significant relationships found were linear and inverse between both IRT and TRT and MO_2_, that is, the more severe the impairment of MO_2_, the thicker the retina. Retinal thickening has been observed previously both in patients with retinal arterial occlusions^41^ and in experimental retinal ischemia^42,43^. It is thought predominately to be the result of cytotoxic edema related to cell volume increases rather than to vasogenic edema related to blood-retinal barrier breakdown^59–61^. The regulation of cell volume is related to sodium transport, and this has been shown to account for about half of the energy used by the retina^62^. Since the cellular functions that are most energy-dependent would tend to be impaired preferentially in ischemia, it makes sense that cytotoxic cellular edema and ensuing increased IRT and TRT would be associated with retinal ischemia-induced reduction in MO_2_. In fact, in the brain, which is similar to the retina, it is known that shortly after ischemia there is marked Na+ influx, K+ efflux and excessive water entry into the intracellular space associated with failure of the Na+/K+ -ATPase pump resulting in cytotoxic edema^63,64^. Nonetheless, there is a paucity of information about the relationship between retinal thickness and MO_2_ in retinal ischemia. Accordingly, our findings are of interest because they provide empirical support for the role of nonperfusion-induced reduction in MO_2_ in the development of retinal thickening in the context of acute nonperfusion. Furthermore, once there is retinal thickening, MO_2_ can be further impaired (and retinal thickening exacerbated) by increases in the distance for oxygen to diffuse from the vasculature to the cells. The lack of a significant relationship between ORT and MO_2_ presumably is explained by the minimal contribution of the retinal vasculature to the oxygen supply of the outer retina.

Our results of retinal thickening and reduced MO_2_ are in agreement with other retinal assessments that were obtained under similar conditions. Histopathology in rats 30 minutes after 90 minutes of ischemia revealed marked edema^43^, and observations obtained 240 minutes after 60 minutes of ischemia showed edema, cytoplasmic vacuolization and condensed nuclei^42^. Fluorescein angiography performed on rhesus monkeys shortly after 135 minutes of ischemia showed only mild leakage^65^. These results are consistent with the predominance of cytotoxic edema with disordered cellular ionic transport secondary to the decreased MO_2_^62^. Electroretinography performed within several minutes after 105–120 minutes of ischemia in rhesus and squirrel monkeys showed a small amount of improvement shortly after reflow, and improvement still was not complete after more than two additional hours^66,67^. Tissue obtained after the effects of an interval of ischemia of about  two hours duration had had time to develop revealed substantial damage on histopathology^16,20,23,67^.

This study had limitations. First, as with all animal experiments, the results in rats may not be generalizable to other species. Second, the OVO method induced nonperfusion in both the retinal and choroidal circulations, whereas in clinical retinal vascular occlusions the choroid is not involved. However, OVO does mimic carotid occlusive disease and ophthalmic artery occlusion, and it likely resembles retinal vascular occlusion in some respects. Third, the calculation of PO_2_ from phosphorescence lifetime used oxyphor constants that were derived from *ex vivo* experiments, which may be different from those under *in vivo* conditions. This may affect absolute PO_2_ values, but not the results of comparing measurements between fellow eyes. Fourth, we did not measure the pH of the blood, which can be reduced in ischemia. Lowering the pH shifts the oxygen hemoglobin dissociation curve to the right and causes SO_2_ to become lower for a given PO_2_ value. While our SO_2_ and O_2_ concentration values may be somewhat high, it is unlikely that this would have a major impact on the overall relationships among the oxygen-related variables. Fifth, our measurements of MO_2_ and OEF assumed that the retinal circulation supplied the same tissue with and without nonperfusion. Future studies are needed to measure retinal tissue PO_2_ in order to document accurately the retinal and choroidal vascular sources of oxygen before, during and after OVO.

In conclusion, immediately after a substantial interval of retinal nonperfusion, the improvements of F and MO_2_ were partial, but greater than that of DO_2_, which led to an elevation of OEF. Furthermore, reduction in MO_2_ was related to thickening of the retina. Since these variables reflect how much energy is available to be utilized by the retina for cellular maintenance and visual processing immediately after an ischemic interval, they may prove to be of great value for clarifying the pathophysiology of nonperfusion retinal injury, predicting tissue outcome and identifying a window of opportunity for clinical intervention to limit retinal damage.

## Methods

### Animals

The study was performed in 17 Long Evans pigmented rats (314–590 g). All animals were treated in accordance with the ARVO Statement for the Use of Animals in Ophthalmic and Vision Research, and the study was approved by the Animal Care Committee at the University of Illinois at Chicago. The rats were anesthetized with intraperitoneal injections of ketamine (100 mg/kg) and xylazine (5 mg/kg) and were given additional doses to maintain anesthesia as needed. The femoral artery was cannulated and a catheter was attached. Both pupils were dilated with 2.5% phenylephrine and 1% tropicamide. Ocular nonperfusion was induced using OVO, and the surgical procedure was performed according to Lafuente and co-workers^20^. Briefly, a longitudinal midline incision over the cranium was performed, and the right orbit was entered subperiosteally to expose the optic nerve. A slit was made in the meningeal sheath, and the exposed optic nerve was gently displaced through the slit. An 8–0 nylon suture was passed between the optic nerve and the meningeal sheath, which contains the ophthalmic vessels^68^. The suture then was passed the rest of the way around the sheath and tied tightly with a slip knot to ligate the vessels. The rats were placed on an animal holder that incorporated copper tubing perfused with heated water to maintain the body temperature. A glass cover slip with 1% hydroxypropyl-methylcellulose was applied to the corneas to minimize their refractive powers and prevent dehydration for imaging. The retina of the eye with OVO was imaged every 15 minutes to document nonperfusion as evidenced by visualization of extremely narrowed arteries and/or segmented blood columns and a pale optic nerve head. After 120 minutes, the ligature was released. Imaging was performed on both eyes of the rats within 20 minutes of release of the ligature (10 minutes of imaging required per eye). The fellow eye served as a paired control. As described elsewhere^40^, an oxygen-sensitive molecular probe, Pd-porphine (Frontier Scientific, Logan, UT), was administered through the femoral arterial catheter (20 mg/kg) for PO_2_ imaging, and 2-µm polystyrene fluorescent microspheres (Invitrogen, Grand Island, NY) were injected through the catheter for retinal blood flow imaging.

### Imaging

Retinal blood flow was measured using our previously reported imaging system^40^. Measurements were made between one half to one optic disc diameter from the edge of the optic disc. Red-free retinal images were analyzed to determine retinal arterial (D_Aind_) and venous (D_Vind_) diameter for each major vessel. Image sequences that displayed motion of fluorescent microspheres were analyzed to provide measurements of blood velocity (V_ind_) in each major vein. F was calculated by the product of V_ind_, π and (D_Vind_)^2^/4, summed over all major veins. For each rat, taking the mean of D_Aind_, D_Vind_, and V_ind_ gave D_A_, D_V_, and V, respectively.

Retinal vascular PO_2_ was measured using our previously reported phosphorescence lifetime system^69^. Briefly, a modulated laser slit at 532 nm wavelength was projected at an oblique angle on the retina for excitation of the oxyphor. A high pass filter (>650 nm) was placed in the phosphorescence imaging path. An electron multiplied intensified charge-coupled device camera with modulated sensitivity acquired phase-delayed optical section phosphorescence images. Images were analyzed to determine phosphorescence lifetime with a frequency domain approach, and PO_2_ in each major artery (PO_2Aind_) and vein (PO_2Vind_) surrounding the optic nerve was calculated by the Stern-Volmer equation^69^. A mean PO_2A_ and PO_2V_ was calculated for each eye. O_2A_ and the oxygen concentrations in the veins (O_2V_) were calculated from PO_2A_ and PO_2V_ respectively using the oxygen-hemoglobin dissociation curve in rats^70^ and values of hemoglobin measured in previous experiments (13.8 g/dL). DO_2_ was calculated by F * O_2A_, and MO_2_ was calculated by F * (O_2A_ − O_2V_). OEF was calculated as MO_2_/DO_2_.

Retinal thickness was measured in a region nasal to the optic nerve using a commercially available spectral-domain optical coherence tomography instrument (Spectralis, Heidelberg Engineering). The instrument’s commercial software was used to automatically measure IRT from the inner limiting membrane to external limiting membrane, ORT from external limiting membrane to Bruch’s membrane, and TRT from internal limiting membrane to Bruch’s membrane.

### Data Analysis

Data were compared between eyes with OVO and fellow eyes using paired t-tests. The relationships of IRT, ORT and TRT with MO_2_ were determined by mixed linear models with individual rats considered as a random variable. Statistical analyses were performed using SSPS Statistics, Version 24 (IBM Armonk, New York). Significance was accepted at P ≤ 0.05.

## Data Availability

The datasets generated during and/or analyzed during the current study are available from the corresponding author on reasonable request.
